# Supplementary material for: Reversible flexoelectric domain engineering at the nanoscale in van der Waals ferroelectrics
Source: Nat Commun. 2024 May 29;15:4556. doi: 10.1038/s41467-024-48892-z (PMC11136971; doi:10.1038/s41467-024-48892-z)
Supplement: Supplementary file 1 — Supplementary Information [file 41467_2024_48892_MOESM1_ESM.pdf]

Supporting Information for

"Reversible flexoelectric domain engineering at the nanoscale in van der Waals ferroelectrics"

Heng Liu[1,2,3, #], Qinglin Lai[1,2,3, #], Jun Fu[1,2,3], Shijie Zhang[4,5], Zhaoming Fu[4,5], and Hualing Zeng[1,2,3, *]

1. International Center for Quantum Design of Functional Materials (ICQD), Hefei National Research Center for Physical Sciences at the Microscale, University of Science and Technology of China, Hefei 230026, China.

2. CAS Key Laboratory of Strongly Coupled Quantum Matter Physics, Department of Physics, University of Science and Technology of China, Hefei, Anhui 230026, China.

3. Hefei National Laboratory, University of Science and Technology of China, Hefei 230088, China.

4. College of Physics and Electronic Information, Yunnan Normal University, Kunming 650500, China.

5. Yunnan Key Laboratory of Opto-Electronic Information Technology, Kunming 650500, China.

* Corresponding author: hlzeng@ustc.edu.cn
# Contributed equally to this work

Supplementary Notes

Supplementary Note 1: Simulation of tip-induced flexoelectric effect in CIPS

Our first-principles simulations were performed by the Vienna ab initio simulation package (VASP)[1–3] with the Perdew–Burke–Enzerhof (PBE) functional[4] and projector-augmented wave (PAW) potential[5]. The cutoff energy for the plane-wave basis was 500 eV, and we used a 3×1×1 Monkhorst-Pack k-point mesh for all models. For calculations of structural relaxation, the convergence criteria for force and energy were set to 0.03 eV/Å and 10^{-6} eV, respectively.

To further understand the microscopic effects of tip imprinting, a simple model was constructed (see Fig. S1a). We used a monolayer of CIPS to simulate the CIPS thin film and capped carbon nanotubes to simulate the probe. The capped carbon nanotube is chemically stable and therefore cannot form bonds with CIPS or cause possible charge transfer, which guarantees that the interaction between the "probe" and CIPS is attributed only to van der Waals (vdW) forces being in accordance with experiments. In addition, hollow nanotubes can reduce the number of atoms in models, which is beneficial for enhancing computational convergence and the convergence rate. This approach is useful for the calculations of large systems. To simulate the process of tip imprinting, we fix two CIPS unit cells on the right and left sides of our model (see the shadowed sections). Simultaneously, we move the carbon nanotube downward to generate the effects of tip imprinting on the CIPS, where the carbon nanotube is also fixed to optimize the structure of the unfixed part (see the dashed ellipse in Fig. S1a). Three different distributions of Cu ions are taken into account in the same bending configurations (see Fig. S1b-1d), corresponding to the same downward depth of the carbon nanotube. The total energies of structural relaxation are calculated. The distribution of Cu ions in Fig. S1d has the lowest energy, indicating that the Cu ions just below the probe and on either side of the probe prefer upward polarization, which is in good agreement with our experiments.

To determine the mechanism of anomalous polarization that occurs under tip pressure, it is intriguing and meaningful to study the deformed geometry of CIPS under tip imprinting. The geometric shape of the bending CIPS can be determined by the position of all the In ions. As shown in Fig. S1e, the black dots are the position coordinates (x and z) of the In ions, where x is the coordinate along the x-axis and z is the height. Therefore, we use the position coordinates to fit the function curve and find that they can be perfectly covered by the Gauss-type function.

$$z(x) = z_0 + A\mathrm{e}^{-\frac{(x-\mu)^2}{2\sigma^2}}, \quad (1)$$

where z_0 and A are constants. The deformation region can be divided into two parts, with the corresponding curvatures being convex and concave. The convex region

generates an upward flexoelectric field, while the concave curvature corresponds to a downward flexoelectric field, potentially revealing the opposite polarization configuration.

For a more comprehensive understanding, a detailed analysis of the flexoelectric field distribution is performed. To simplify the calculation, the focus is directed toward the lower segment, which can be represented by an arch model (see schematic in Fig. S3). For thin flakes with a certain thickness *d*, the upper part experiences compressive strain upon bending, while the lower part undergoes tensile strain. Thus, the strain gradient *∂u/∂z* is perpendicular to the surface and can be written as[6]

$$\frac{\partial\mu}{\partial z} = \frac{\mu_2-\mu_1}{d} = \frac{\frac{\left(R+\frac{d}{2}\right)\cdot\frac{l_0}{R}-l_0}{l_0}-\frac{\left(R-\frac{d}{2}\right)\cdot\frac{l_0}{R}-l_0}{l_0}}{d} = \frac{1}{R} = \kappa\,, \tag{2}$$

where κ represents the curvature. Equation (2) depicts the relationship between the strain gradient and the curvature κ. The curvature is mathematically defined as

$$\kappa = \frac{|z''(x)|}{(1+z'(x)^2)^{3/2}}, \tag{3}$$

where $z(x)$ represents the height. This formula solely quantifies the magnitude of the curvature without providing any information about the concavity or convexity of the curvature. Consequently, this approach solely characterizes the magnitude of the strain gradient while neglecting to specify its direction. In flexoelectric physics, we propose the concept of "two-way curvature" κ_t.

$$\kappa_t = \frac{z''(x)}{(1+z'(x)^2)^{3/2}}, \tag{4}$$

where the sign and value of κ_t describe the direction and magnitude of the strain gradient, respectively. Hence, for a known deformation, $z(x)$, κ_t can be effectively utilized to quantify the corresponding strain gradient and flexoelectric field, which is essential for advanced studies involving flexoelectric effects. Taking (1) into equation (4), the calculated two-way curvature of the Gauss function is written as follows:

$$\kappa_t(x) = \frac{\frac{A}{\sigma^2}\exp\left[{-(x-\mu)^2}/{2\sigma^2}\right]\left(1-\frac{(x-\mu)^2}{\sigma^2}\right)}{\left[1+A^2\frac{(x-\mu)^2}{\sigma^4}exp\left[{-(x-\mu)^2}/{\sigma^2}\right]\right]^{\frac{3}{2}}}. \tag{5}$$

The corresponding curve is given in Fig. S1f.

Notably, the signs of the two-way curvature exhibit opposition both below and on either side of the probe, implying the existence of opposing flexoelectric fields. This observation offers a credible rationale for the occurrence of two-way polarization induced by tip imprinting in experimental studies.

Supplementary Note 2: Calculated in-plane anisotropic flexibility in the CIPS

To clarify the anisotropy of polarization caused by tip imprinting, we constructed a periodic corrugated structure (see Fig. S2), which was used in our previous work[7] and was confirmed to be effective at describing the flexoelectric effects. The degree of bending strain is controlled by changing the scale factor (λ) of the supercell size, which is defined by the formula $\lambda=1-t/l_0$. Here, l_0 is the length of the flat supercell, and t is the length of a corrugated supercell that includes the same number of unit cells (see ref. [7]). In this way, stable corrugated structures can be obtained through the structural relaxation of all atoms.

We construct two corrugated models by considering two nonequivalent crystal orientations (see Fig. S2a). The models in Fig. S2b and 2c correspond to <100> and <1-10> orientations, respectively, which have the same length (see Fig. S2d and 2f) and the same number of primitive cells (8 cells). We use the method mentioned above to construct the corrugated models (see Fig. S2e and 2g). The two corrugated models have the same values of λ, which means that they have the same bending degrees. Therefore, we can compare the energy changes between straight and corrugated structures to justify the flexibility of the two crystal orientations. The calculated results suggest that bending along the <1-10> orientation has a lower deforming energy, which indicates that the <1-10> surface deforms more under the same loading force. This may explain the emergence of the tri-petal domain in CIPS, which is the manifestation of lattice symmetry and anisotropy at the macroscopic scale.

Supplementary Note 3: The PDOS of Cu in flat and curving CIPS

The effects of curving on the electronic structures are also studied. We focus on the Cu ions in the upward and downward strain areas, where the value of the curvature is the

largest. Therefore, it is natural that the electronic structures of these Cu ions could significantly change. For comparison, the PDOSs of Cu in flat and curved CIPSs were calculated (as shown in Fig. S3a and S3b, respectively). The dz^2 orbital of Cu significantly changes. In the flat model, the PDOS of the dz^2 orbital (red curve) is characterized mainly by multiple peaks. In contrast, in the curved model, there is only one main peak, which becomes sharper than that in the flat model. At the same time, other peaks shrink remarkably or vanish. These results indicate that the curving causes the dz^2 orbital of Cu in the strain area to be more localized. This change in the electronic structure is understandable. The spatial orientation of the dz^2 orbital is out-of-plane and perpendicular to the CIPS film. Therefore, in the curved model, the out-of-plane bending strain (upward and downward) has a large effect on the dz^2 orbital. Furthermore, the localization of dz^2 orbitals can be attributed to the additional polar displacement of Cu ions along the out-of-plane direction due to the flexoelectric effect in the curve model.

Supplementary Note 4: Details of the plastic and elastic substrate preparations

The flexible substrate was made by spin coating PMMA onto a silicon substrate. As shown in Fig. S5. First, 5 nm titanium (Ti) and 30 nm gold (Au) were evaporated onto a flat silicon substrate via e-beam evaporation to enhance the adhesion of PMMA. c, the PMMA solution (4%/10%) was spin-coated (6000 rpm/8000 rpm, 40 s) on the gold film on the silicon substrate and heated on a hot plate at 160 °C for 30 s. Finally, 10 nm of Au was evaporated on the surface of the PMMA as a bottom electrode to ensure the operation of the PFMs. The CIPS flakes were obtained by mechanical exfoliation and transferred onto a flexible substrate via the all-dry transfer technique. Following this, the sample and substrate underwent deformation induced by the loading force using an atomic force microscopy (AFM) tip nanoimprinting technique. Utilizing PMMA at a 4% concentration resulted in the persistence of deformation after the removal of the probing tip. In contrast, the use of a more elastic PPMA at a 10% concentration led to the restoration of flatness within a constrained range of deformation.

Supplementary Note 5: Details of the nanoimprinting technique

Our indentation tests utilized four types of nanoimprinting tips: an RTE-SPA-300, a SSS-NCHR, a Biosphere-N20, and a Biosphere-N100. As depicted in Fig. S8a-8d, the indentations created by triangular tips exhibit a triangular shape, while those created by spherical tips display a circular morphology, indicating a direct correlation between deformation and tip morphology. The impact of different loading forces on the deformation and manipulation stability of the different tips was investigated, as shown in Fig. S8e-8h, with the loading force gradually increasing from 60 nm to 140 nm. The depth of the indentations was directly proportional to the applied force. The error bars indicate that Biosphere-N100 exhibited the highest stability. The corresponding flexoelectric field induced by this tip is nearly proportional to the applied force, as shown in the inset of Fig. S8h. Nonetheless, under the same loading force, the substrate's elasticity and the sample's thickness have a vital influence on the deformation, which is directly related to the flexoelectric field.

It should be noted that the sample thickness and the substrate hardness strongly impact the tip deformation in the tip imprinting technique. Therefore, the dependence of the deformation depth and the applied force must be investigated before performing the tip imprinting technique. As shown in Fig.S9, the relationship between deformation depth and the applied force on substrates of 4% PMMA with a 10 nm gold (Au) layer, for samples with the thicknesses of 8, 16, 18, and 23 nm, was investigated using a Bio-SphereN-100 imprinting tip. It is clearly found that the tip-induced deformation depth increases proportionally with the applied force for the same sample thickness, while thinner samples exhibit greater deformation when subjected to the same force. Therefore, for a uniform expression, using the depth of deformation to describe the effect of the varied flexoelectric field on ferroelectric domains is more reliable. Furthermore, the use of spherical tips minimized the influence of tip morphology on domain manipulation. Consequently, Biosphere-N100 was chosen for subsequent experiments, and its topography is presented in Fig. S10.

To further quantify the tip-induced unconventional flexoelectric effect in ultrathin ferroelectrics, we investigated varied imprinting forces on flexoelectric field, as

depicted in Fig. S11. Fig. S11a demonstrates that the depth of deformation linearly increases from 5 nm to 20 nm as the loading force increases from 3 μN to 6 μN, and the corresponding strain gradient is shown in Fig. 3b. The flexoelectric field can be calculated using Equation (3) in the main text and is sequentially shown in Fig. S11c-11f. Notably, the maximum E_{flexo} increases from 2.28×10^6 V/cm to 4.13×10^6 V/cm and from -5.21×10^6 V/cm to -10.27×10^6 V/cm. The theoretical threshold for the transition to high polarization states is approximately 10^7 V/cm. Moreover, it has been demonstrated that in-plane tensile strain can reduce the coercive field. As depicted in Fig. S11b, the deformation can be approximated as a triangle for simplification, and the in-plane strain is expressed as

$$s = \frac{2\sqrt{(\frac{l_0}{2})^2+h^2}-l_0}{l_0} \times 100\% \ , \tag{6}$$

where l_0 is the original length and the depth of the indentation is h. Therefore, the geometry with l_0 = 160 nm and h = 20 nm results in a maximum in-plane strain of approximately 3.07%. This further reduces the coercive field, leading to the observation of high polarization states, as shown in Fig. S12.

Supplementary Note 6: Change of the surface roughness

To further evaluate the increase in surface roughness caused by the tip imprinting technique, we performed a series of repeated imprinting experiments (1 to 15 cycles) on the same sample area. As shown in Fig. S16 in the revised supporting information, the morphological roughness slightly increases with the number of cycles. These results indicate that the demonstrated transient two-way flexoelectric control is noninvasive and safe for maintaining the sample morphology.

Supplementary Figures

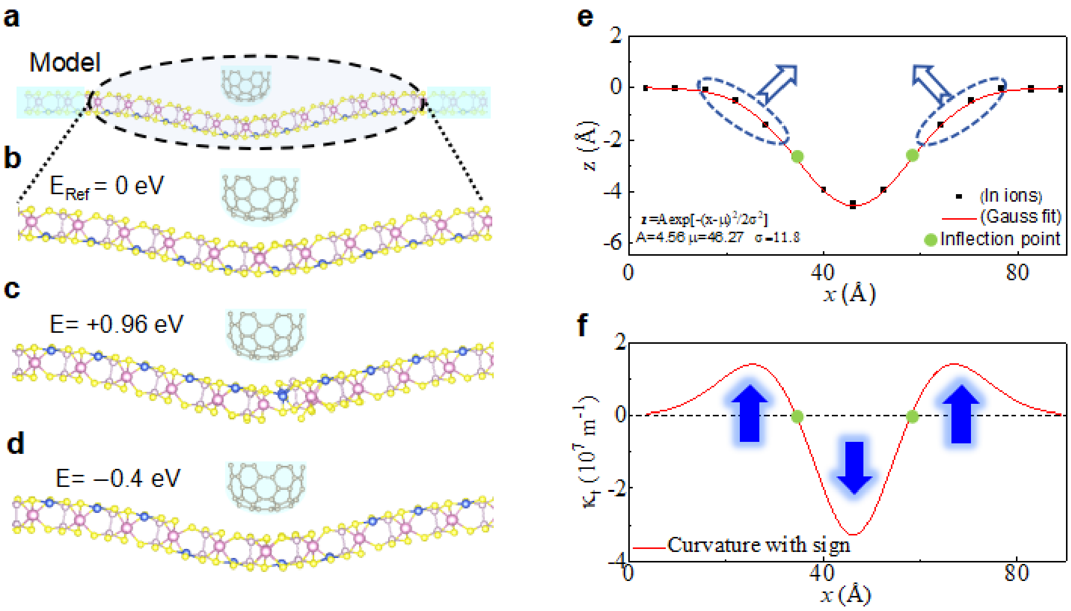

Supplementary Fig. 1: Simulation of the tip-induced flexoelectric effect in CIPS. a Bending configuration of CIPS caused by nanotip imprinting. **b-d** Three different distributions of Cu ions in the CIPS lattice with the same bending degree. In all three configurations, the positions of all the atoms are relaxed. By comparing their energies, it was found that the flexoelectric effect leads to a certain polarization of the CIPS under tip imprinting. The energy for placing all the Cu ions downward corresponds to zero potential energy. **e** The geometric shape of the bending CIPS. For simplicity, the bended CIPS here is represented by the positions of the relaxed In atoms. The deformation is well fitted by a Gauss-type function. From the second derivatives of the Gaussian fitting, we find inflection regions of convex and concave curvatures, as indicated by the blue dashed lines and arrows. **f** The calculated two-way curvatures in the bent CIPS. The positive and negative values denote upward and downward strain gradients, respectively.

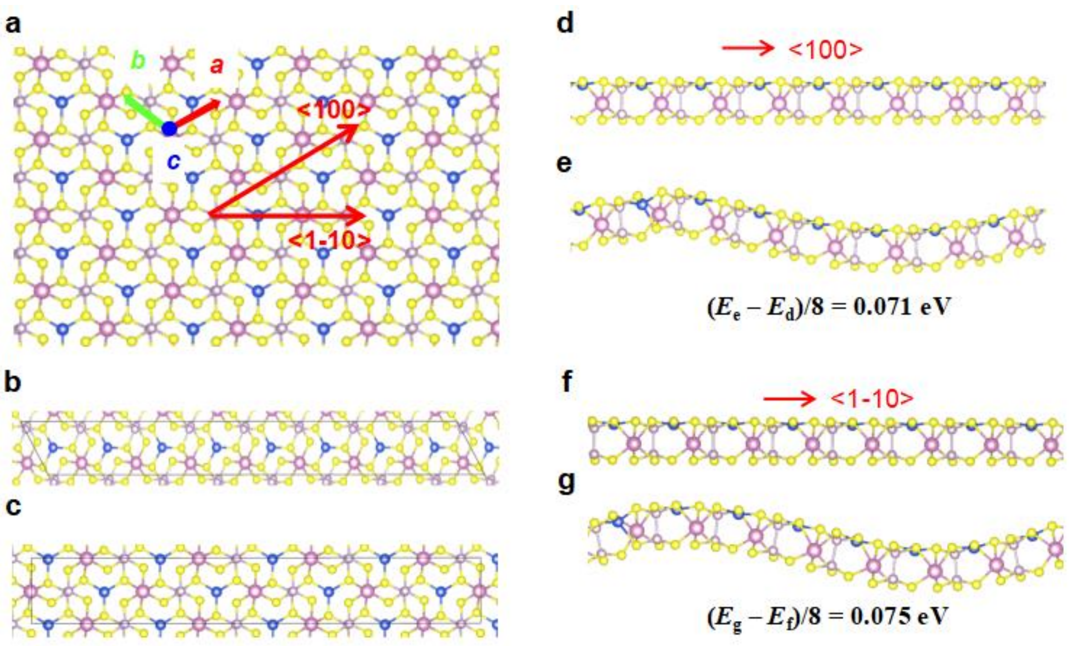

Supplementary Fig. 2: Calculated in-plane anisotropic flexibility in CIPS. a Crystal structure of single-layer CIPS. **b-c** Models correspond to <100> and <1-10> orientations, respectively. **d-g** Deform equal lengths of CIPS to the same depth along <100> and <1-10> directions, respectively, and calculate the energy difference of strain and non-strain CIPS along <100> and <1-10> orientation, respectively.

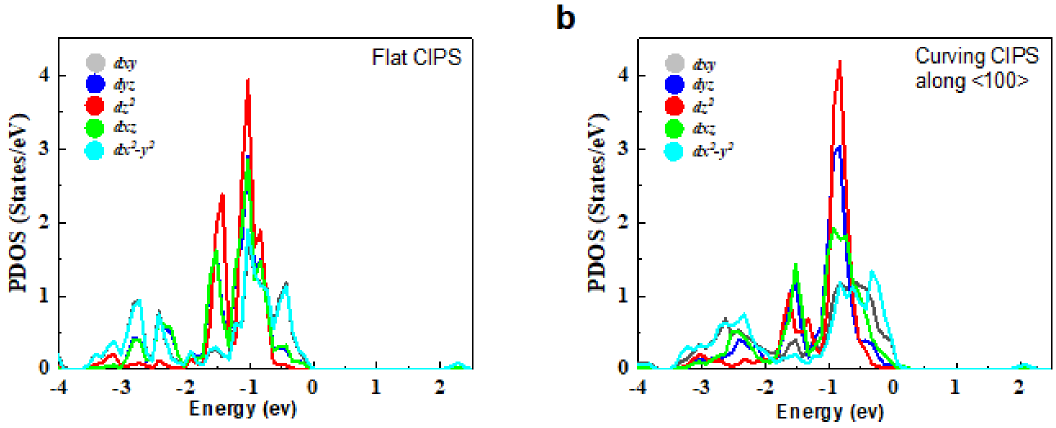

Supplementary Fig. 3: The PDOS of Cu in flat and curving CIPS. a PDOS of Cu in a flat CIPS. **b** PDOS of Cu in a curved CIPS <100>.

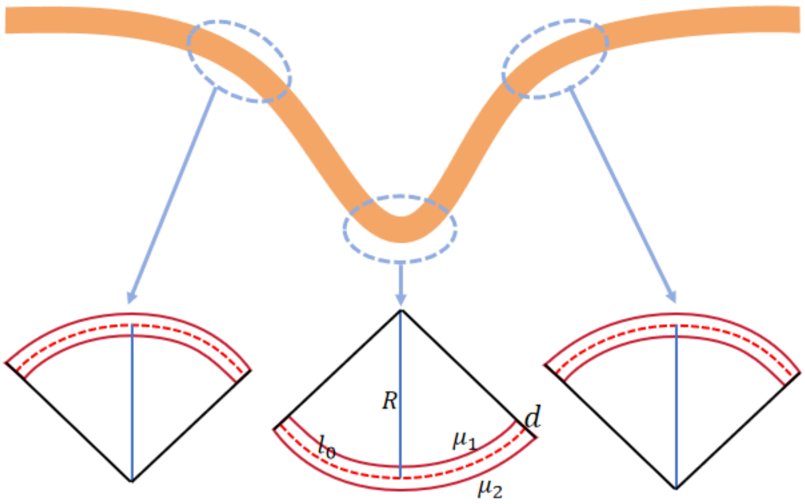

Supplementary Fig. 4: Bending model for the strain gradient calculation. *d* represents the thickness of the film, l_0 represents the initial length, u_1 represents the strain on the upper surface, u_2 represents the strain on the lower surface, and *R* represents the radius of curvature.

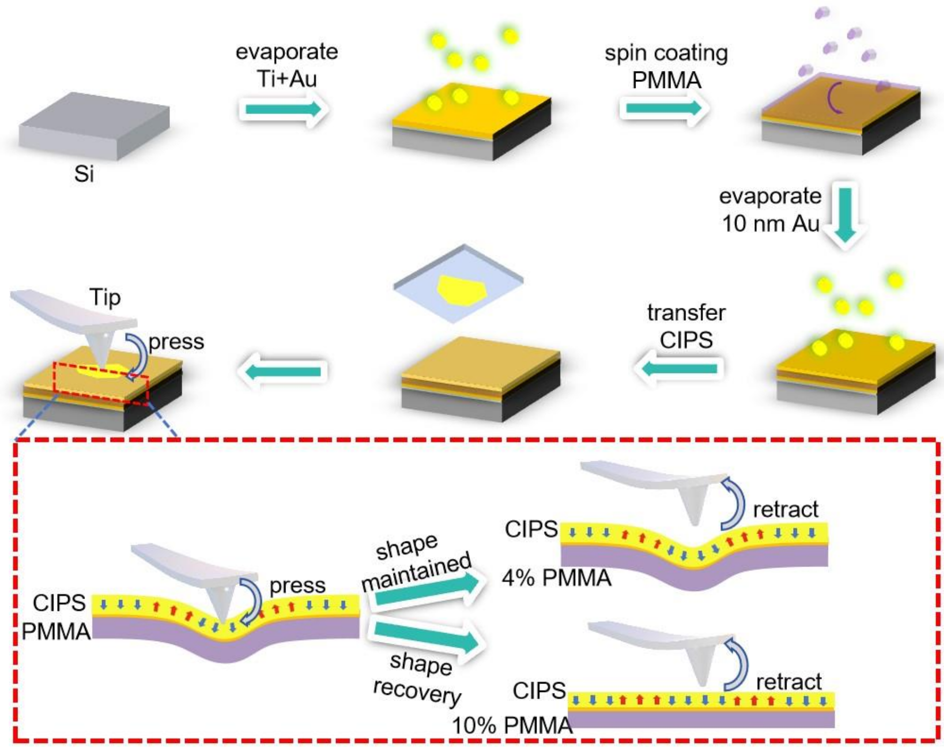

Supplementary Fig. 5: Flow diagram of the sample preparation process on flexible substrate. The zoomed figure shows the nano-tip imprinting technique in shaping and transient flexoelectric control.

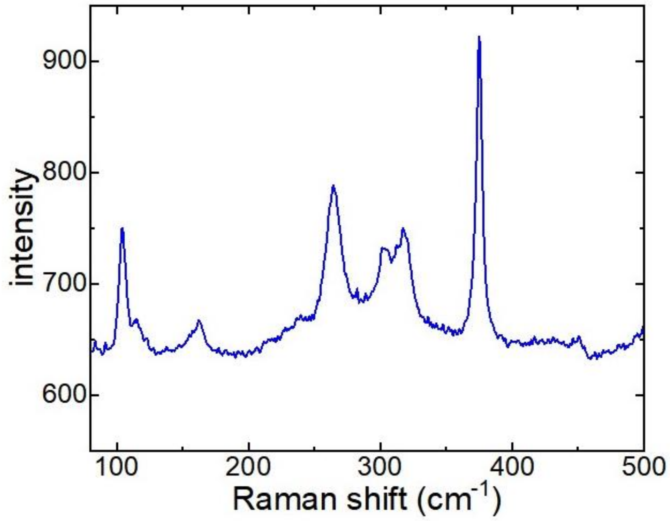

Supplementary Fig. 6: Raman spectrum of a 22 nm CIPS flake. The characteristic Raman modes were observed at 275, 325, and 384 cm^{-1}.

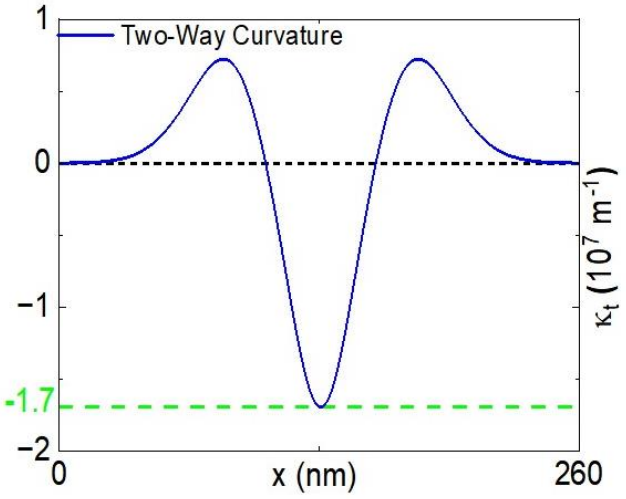

Supplementary Fig. 7: The corresponding strain gradients for Fig. 2f. The positive and negative κ_t values indicate the presence of opposite strain gradients in the imprinted region, which correlate well with the ferroelectric polarization shown in Fig. 2i.

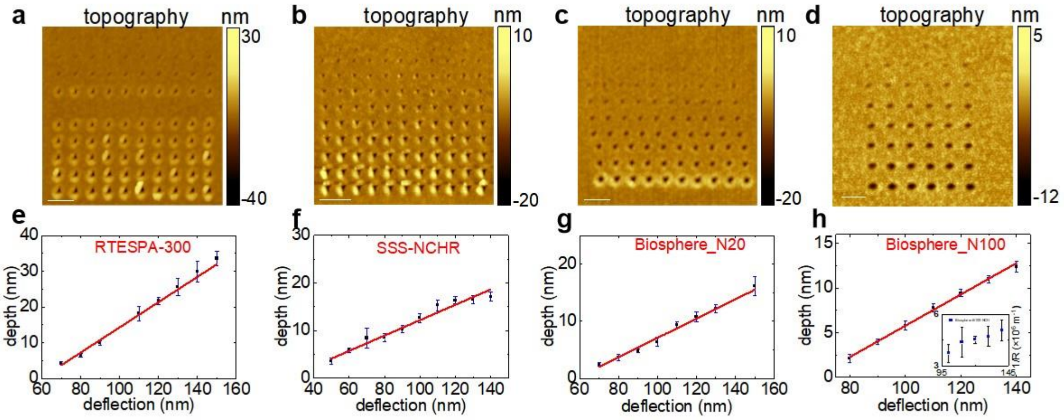

Supplementary Fig. 8: Indentation tests with different tip forces. a-d Morphology after imprinting with RTE-SPA-300, SSS-NCHR, Biosphere-N20, and Biosphere-N100 tips at a loading deflection of 60-140 nm. **e-h** Relationships between the depth of deformation and loading force. The figure shows that the depth deformation is directly proportional to the applied loading force and is unrelated to the geometry and size of the tip. The error bars indicate the height variations for holes in each figure.

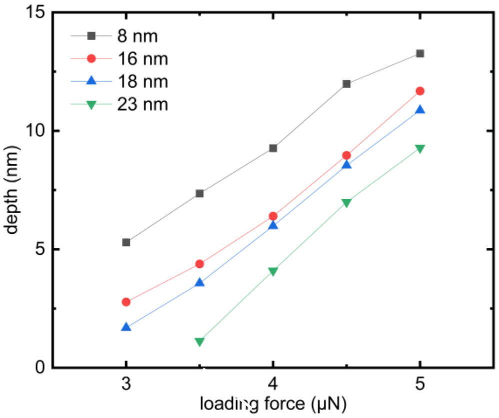

Supplementary Fig. 9: Dependence of the deformation depth on loading force. The tip-induced deformation depth is directly proportional to the applied loading force for the same thickness. Conversely, under the same loading force, the thicker the sample, the smaller the deformation.

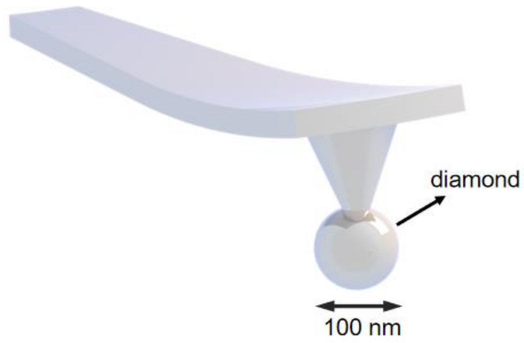

Supplementary Fig. 10: Schematic of the geometry of AFM tip Biosphere-N100. The tip diameter is 100 nm.

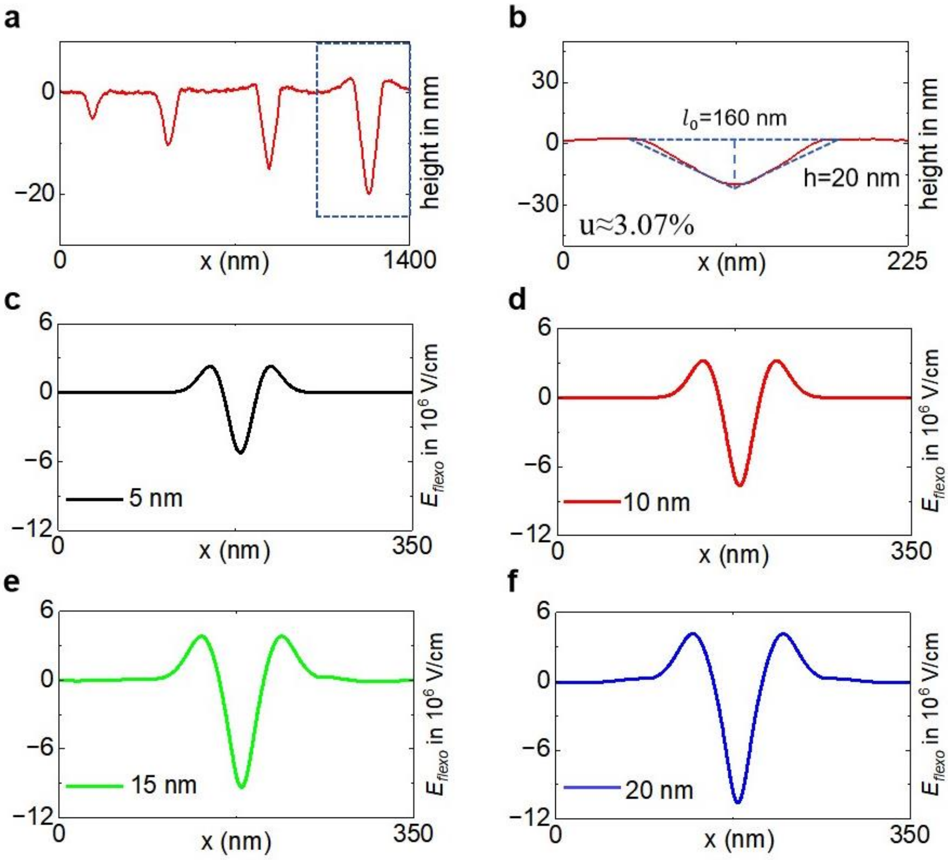

Supplementary Fig. 11: Corresponding strain gradients for the indentations in Fig. 3a. **a** Corresponding height line profile from Fig. 3a. **b** Simple calculation of in-plane strain for an indentation depth of 20 nm. The deformation can be approximated as a triangle as marked by the blue dashed line. **c-f** Spatial distributions of E_{flexo} at depths of 5, 10, 15, and 20 nm, respectively.

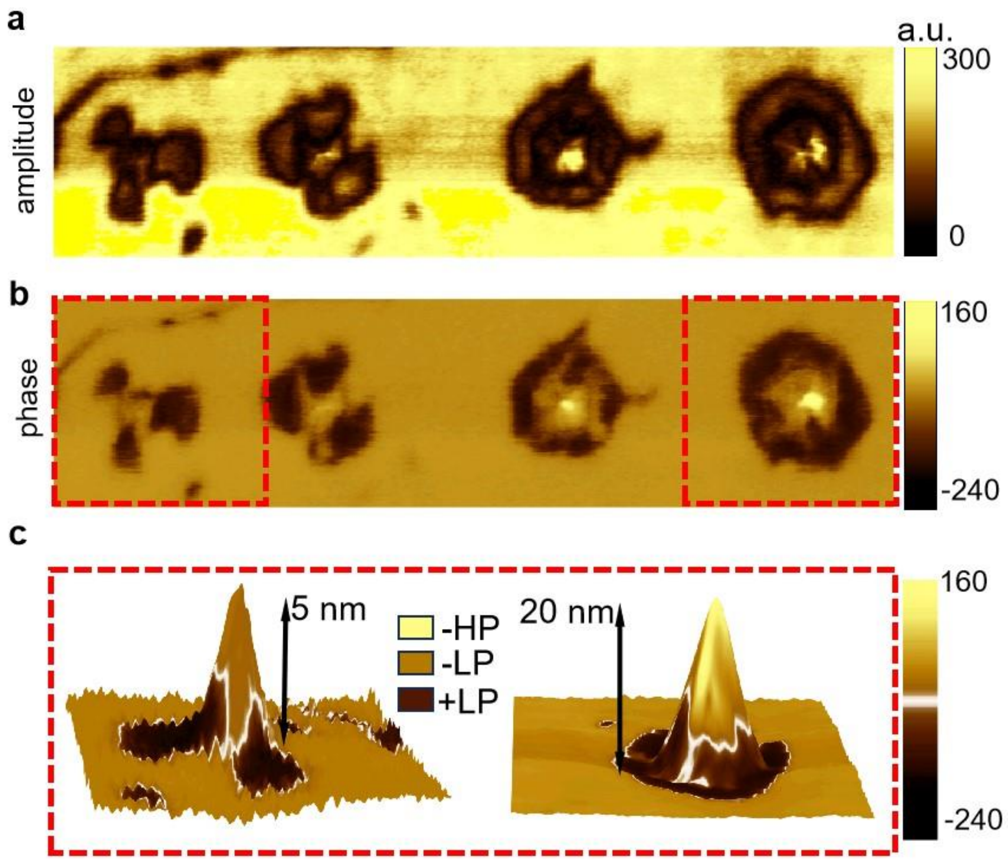

Supplementary Fig. 12: Corresponding domain structures for the indentations in Fig. 3a. **a** Corresponding PFM amplitude and **b** PFM phase images for Fig. 3a. **c**, PFM phase image imposed with 3D AFM topography.

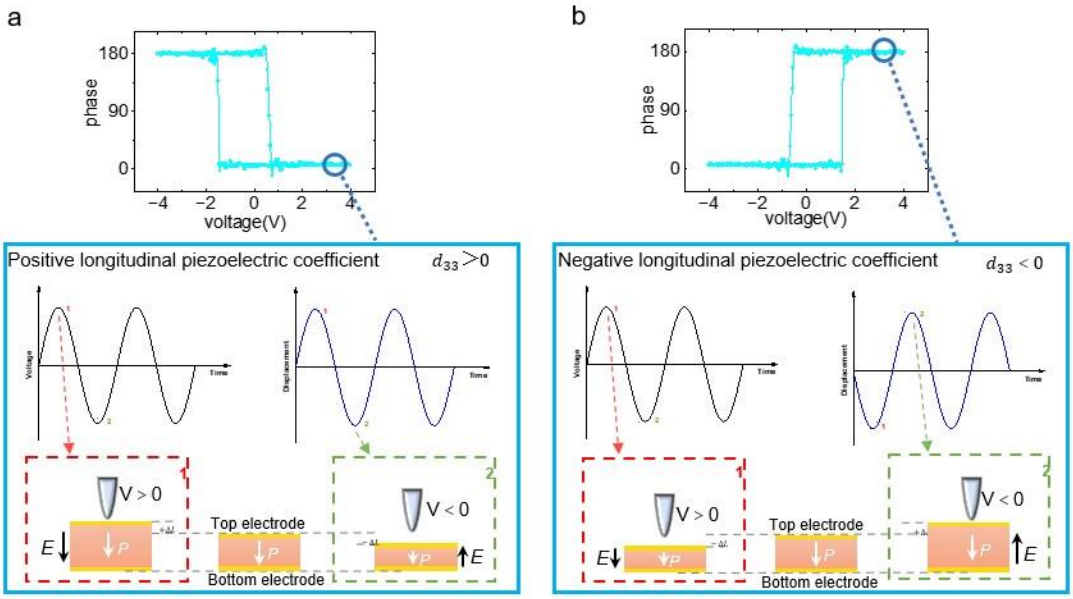

Supplementary Fig. 13: PFM characterization of the high and low polarization states in CIPS. a Piezoelectric response in the high polarization state of CIPS. Upper panel: A representative phase hysteresis loop of CIPS. Lower panel: The high-polarization state corresponds to a positive longitudinal piezoelectric coefficient. When the polarization is downward, the voltage and displacement signals are in phase, corresponding to a 0° phase lag. **b** Piezoelectric response in the low polarization state of CIPS. Upper panel: A representative phase hysteresis loop of CIPS. Lower panel: The low-polarization state corresponds to a negative longitudinal piezoelectric coefficient. When the polarization is downward, the voltage and displacement signals are out of phase, corresponding to a 180° phase lag.

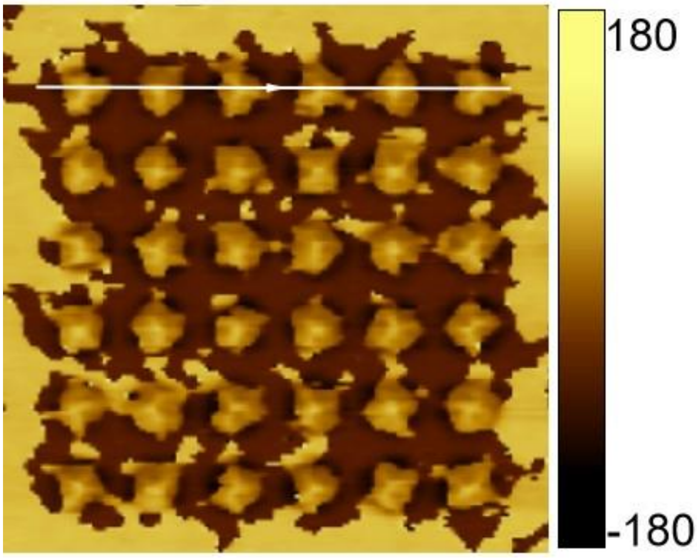

Supplementary Fig. 14: Noninvasive generation of high-density nanodomains. With a 150 nm step resolution, 36 domains are mechanically written in 1 μm^2, equal to a density of 31.4 Gbit/in^2.

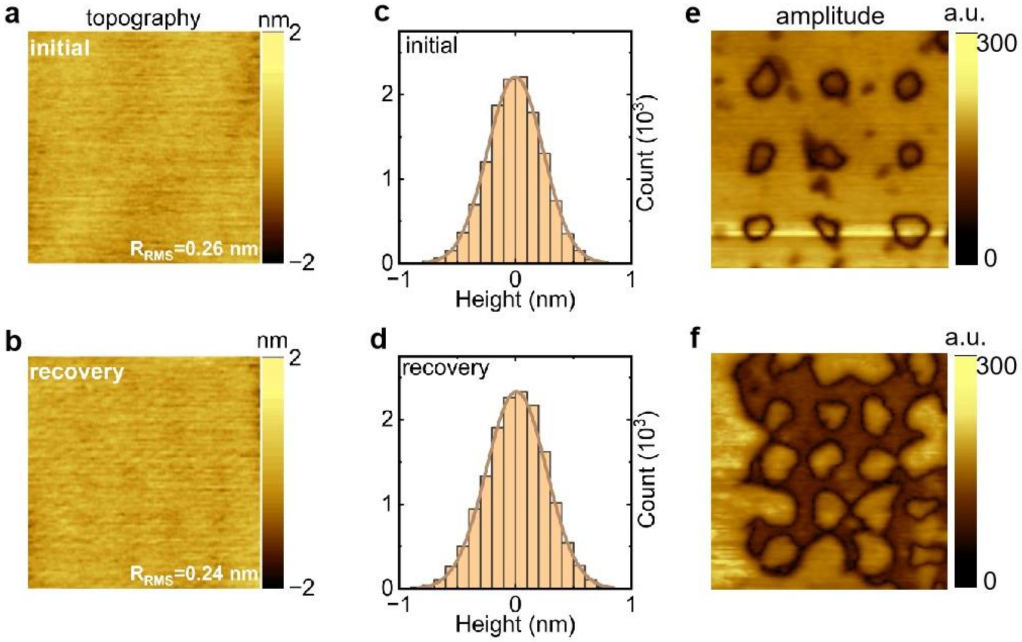

Supplementary Fig. 15: Morphological changes induced by tip indentation. a Initial topography before operation and **b** the final topography after transient flexoelectric control operations, respectively. **c-d** Statistical histograms on the height variations of the topography before and after the operation. **e-f** Corresponding PFM amplitudes images for the data in Fig. 4e and 4g.

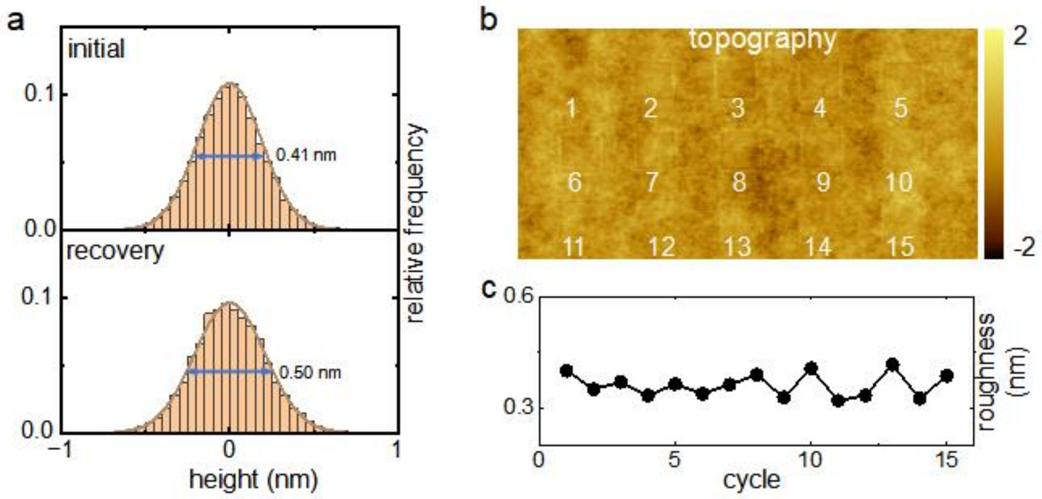

Supplementary Fig. 16: Morphological changes after repeated indentations. a Histograms of the topography before and after the operation. **b,c** The AFM topography and corresponding roughness of repeated imprinting areas from 1 to 15 cycles.

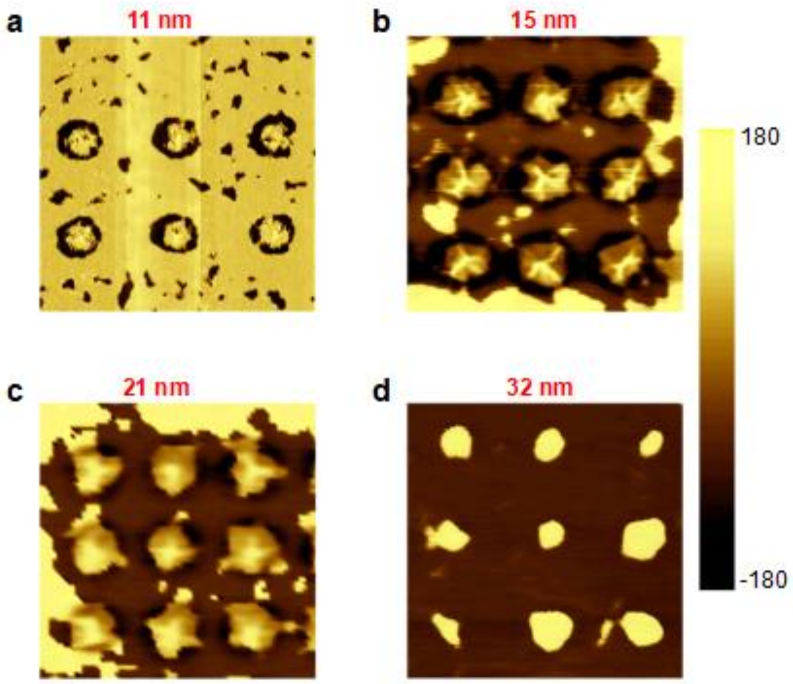

Supplementary Fig. 17: Verification of flexoelectric control in samples with different thicknesses. a-d PFM phase images after tip imprinting in samples with film thicknesses of 11, 15, 21, and 32 nm.

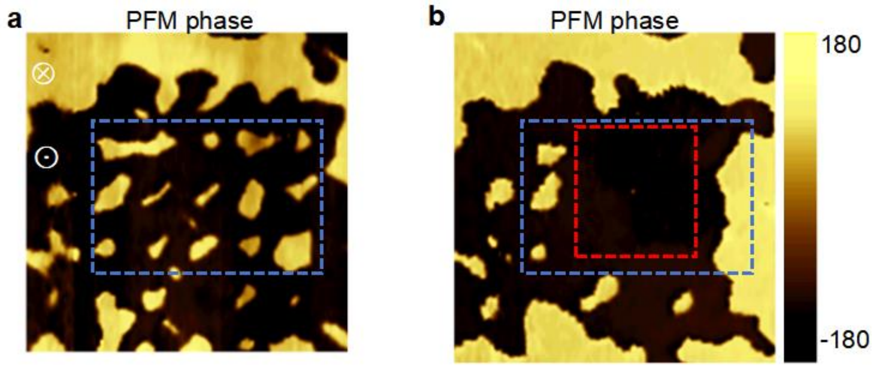

Supplementary Fig. 18: Electrical switch of mechanically engineered domains. a PFM phase image after tip imprinting with a 3×5 array, where the area is delineated by a blue dashed line. **b** PFM phase image after electric writing at -5 V, where the area is delineated by the red dashed line.

References

1. Kresse, G. & Furthmüller, J. Efficient iterative schemes for *ab initio* total-energy calculations using a plane-wave basis set. *Phys. Rev. B* 54, 11169–11186 (1996).
2. Kresse, G. & Hafner, J. Ab initio molecular dynamics for liquid metals. *Phys. Rev. B* 47, 558–561 (1993).
3. Blöchl, P. E. Projector augmented-wave method. *Phys. Rev. B* 50, 17953–17979 (1994).
4. Perdew, J. P. & Zunger, A. Self-interaction correction to density-functional approximations for many-electron systems. *Phys. Rev. B* 23, 5048–5079 (1981).
5. Perdew, J. P. *et al.* Restoring the density-gradient expansion for exchange in solids and surfaces. *Phys. Rev. Lett.* 100, 136406 (2008).
6. Lee, D. Flexoelectricity in thin films and membranes of complex oxides. *APL Materials* 8, 090901 (2020).
7. Chen, C. *et al.* Large-scale domain engineering in two-dimensional ferroelectric $CuInP_2S_6$ via giant flexoelectric effect. *Nano Lett.* 22, 3275–3282 (2022).
